# Advances in real time smart monitoring of environmental parameters using IoT and sensors

**DOI:** 10.1016/j.heliyon.2024.e28195

**Published:** 2024-03-20

**Authors:** T. Lakshmi Narayana, C. Venkatesh, Ajmeera Kiran, Chinna Babu J, Adarsh Kumar, Surbhi Bhatia Khan, Ahlam Almusharraf, Mohammad Tabrez Quasim

**Affiliations:** a Department of Electronics and Communication Engineering, KLM College of Engineering for Women, Kadapa, A.P, 516003, India; b Department of Electronics and Communication Engineering, Annamacharya Institute of Technology and Sciences, Rajampet, 516126, A.P, India; c Department of Computer Science and Engineering, MLR Institute of Technology, Hyderabad, Telangana, 500043, India; d School of Computer Science, University of Petroleum and Energy Studies, Dehradun, Uttarakhand, India; e School of Science, Engineering and Environment, University of Salford, Manchester, United Kingdom; f Department of Electrical and Computer Engineering, Lebanese American University, Byblos, Lebanon; g Department of management, College of Business Administration, Princess Nourah Bint Abdulrahman University, P.O. Box 84428, Riyadh 11671, Saudi Arabia; h Department of Computer Science and Artificial Intelligence, College of Computing and Information Technology, University of Bisha, P.O Box 551, Bisha, Saudi Arabia

**Keywords:** Environmental parameter, Internet of things, Sensors, Smart monitoring, Webpage, Weather station

## Abstract

People who work in dangerous environments include farmers, sailors, travelers, and mining workers. Due to the fact that they must evaluate the changes taking place in their immediate surroundings, they must gather information and data from the real world. It becomes crucial to regularly monitor meteorological parameters such air quality, rainfall, water level, pH value, wind direction and speed, temperature, atmospheric pressure, humidity, soil moisture, light intensity, and turbidity in order to avoid risks or calamities. Enhancing environmental standards is largely influenced by IoT. It greatly advances sustainable living with its innovative and cutting-edge techniques for monitoring air quality and treating water. With the aid of various sensors, microcontroller (Arduino Uno), GSM, Wi-Fi, and HTTP protocols, the suggested system is a real-time smart monitoring system based on the Internet of Things. Also, the proposed system has HTTP-based webpage enabled by Wi-Fi to transfer the data to remote locations. This technology makes it feasible to track changes in the weather from any location at any distance. The proposed system is a sophisticated, efficient, accurate, cost-effective, and dependable weather station that will be valuable to anyone who wants to monitor environmental changes on a regular basis.

## 1 Introduction

The environment can be characterized simply as the total influence of all living and non-living elements on human life. Non-living components include land and water, as well as warm temperatures, stones, and the atmosphere, whereas all living elements include organisms such as plants, woodlands, fisheries, and raptors. Biotic element-related things, especially people of different sections like farmers, sailors, voyagers and mine workers, are regularly connected with ecological variables like temperature, humidity, atmospheric pressure, air-related factors called air quality, CO2, methane-like gases, wind speed and direction, water-related factors called rainfall, pH levels, moisture in the soil, quantity of water, and light quality and turbidity [1]. Commonly used traditional forecasting techniques include trend, persistence, climatology, and analogues of weather systems. All of these techniques rely on a few fundamental presumptions to extrapolate the weather into the future [2]. In the meantime, the world's population has expanded, leading to developments in urbanization, industrialization, agricultural land, and energy utilization. To keep up with increased demand, technological developments that enable precise monitoring of environments are required. Also, due to their ignorance of modern farming practices, Indian farmers are not producing the appropriate amount of food [[3], [4], [5], [6], [7]]. Accurate yield prediction will be aided by predictive analytics, which will require knowledge about the functional relationship between yield and environmental factors, which is delivered in the form of a dataset [8]. Remote sensors are used to monitor environmental characteristics that vary from place to place. The intention for environmental monitoring aims to control and reduce an activity's adverse environmental effects along with the risks of adverse effects on the natural environment, and also to safeguard the health of humans. Environmental monitoring employs methods that discover and analyze environmental conditions to determine the environmental impact of an activity [9,10].

Environmental monitoring is used to observe the overall quality of an environment, environmental factors, and the influence of a specific action. Such types of monitoring practices may be set up to identify irregularities or specific situations and then send alerts by email or text message, as well as activate automated operations. The consistent gathering of measurements and data from our physical surroundings using sensors and connected devices is what IoT-based smart environmental parameter monitoring is all about [11]. Sensors built into agricultural systems, pipelines, tanks, climate stations, oceanic applications, and industrial machinery may record temperature, moisture, water content, water leaks, and various other physical attributes all around the world. Intelligent, interconnected devices with inbuilt connectivity modules are able to analyze that information and immediately send extremely important information to the cloud or a data center for additional action or analysis using edge computing technology. An IoT-enabled environmental monitoring system acts as an application's eyes, ears, and mouthpiece, watching, listening, reporting, and even taking action to prevent damage [12,13].

Modern digital farming employs datasets containing data from decades with numerous parameters required for yield prediction, such as soil type, soil nutrients, soil pH, area of cultivation, production history, and so on. Prediction in traditional farming is based on farmer experience, which is time-intensive and inefficient. As a result, a move from traditional farming to digital farming is required, which is doable with data-driven Internet of Things technology [14,15].

Most researchers never looked into usage other than agriculture or weather monitoring stations in depth. Most modern technologies just use only one of a few sensors, which are again ineffective at collecting any useful data. The accuracy of the sensor also plays a vital role. If multiple sensors are used, then power issues creep into the system. Data analytics can be employed to evaluate features including climate change, variation in rainfall, groundwater resources, variations in human metabolism, and plantations using data from the sensor. The following literature from the same research work is reviewed: In order to get data on an OLED display, Kishore et al. [16] monitored the weather conditions using sensors connected to WeMo and cloud services. ZigBee was used by Saini et al. [17] to connect a PC with a GUI; data was downloaded from the internet and displayed on an LCD screen. To detect temperature and humidity, Palle et al. [18] employed the CC3200, which is a basic Link Wi-Fi internet chip. Three meteorological variables were measured by Shaout et al. [19]: temperature, wind speed, and wind direction. The information was delivered to an LCD. Amber Katyal et al. [20] used an Arduino Uno-based system to find out if the environment had light, cloudy weather or not. Instead of using sophisticated weather observatories, which may perform on a large scale, this device's distinctive feature is that it can be implemented in any critical situation or local region. These also function on public wireless LANs on a smaller scale. A few sensors are employed to find a few parameters and lack the ability to display and send data. Abu Saleh Bin Shahadat et al. [21] created a system for measuring weather forecast conditions with the NodeMCU Board and Blynk IoT technology, which measures meteorological data such as temperature, pressure, humidity, and rainfall. Shubham R. V. et al. [22] developed an IoT-based weather monitoring system using a Raspberry Pi to collect data related to the environment called the atmospheric pressure, rainwater level and light intensity. But they did not mention the display method of the collected information.

R. Kavin et al. [23] introduced a model in which sensors are set up to track factors like temperature, humidity, and CO value, and data is gathered using the IDE (Integrated Development Environment). The final client will receive the outcome evaluation over Wi-Fi. Through every phase of detection, the devices are controlled by an AT Mega 328 controller, which also accumulates sensor information and sends it to end clients via the cloud. Mohit Tiwari et al. [24] suggested a wireless and IoT-based weather observation mechanism. Their systematic development of a framework uses a Wi-Fi module, sensors, and an Arduino UNO board to transfer data to cloud computing offerings. Additionally, a webpage is made that demonstrates all of the information and shows the information to clients. Pasika and Gandla [25] suggested a system for monitoring that comprises a range of sensors to assess different environmental parameters such as viscosity, pH levels, the level of water in the tank, moisture of the surrounding environment, and the temperature of the water. The sensors and the microcontroller unit (MCU) are linked, and the remote monitoring tool (PC or mobile) provides further processing. The collected data will be uploaded to the cloud using the Internet of Things (IoT)-based ThingSpeak application to follow the water quality under test.

Kumar et al. [26] suggested a low-cost, IoT-based solution for monitoring the water's quality at the moment. The system that was developed employs a range of detectors that determine both the physical and chemical characteristics of the water that it contains. A Raspberry Pi processor is associated with many different kinds of sensors, including carbon dioxide (CO2) sensors, pH level sensors, turbid detectors, thermometers, and water depth sensors, in this sophisticated system for monitoring water quality. These sensors oversee the entire process, with monitoring handled by cloud-based wireless communication devices. Gupta et al. [27] offer a model that autonomously examines water's biological attributes such as viscosity, pH level, and temperature. The ESP32 chip was used for underwater communication due to its low power consumption and built-in Wi-Fi. The Internet of Things-connected model was created by combining communication modules called a turbidity detector and an acidity detector (a pH sensor). Using multilevel IoT architecture, Vasanth K. Babu et al. [28] created a system that tracks environmental factors. Utilized various sensors to gather data on various environmental characteristics, which is then sent through SMS or to a webpage. Farmers are given information about the environment, and the experiments' results are summarized. The proposed system is more than just a model for gathering data; it also accesses data and evaluates it to provide farmers with a yield indicator.

The research project demonstrated by Varsha et al. [29] is an effective IoT-based strategy for measuring the quality of water in addition to a comprehensive examination of the different methodologies of water quality monitoring and the origins and effects of water contamination. Despite the existence of numerous superb smart water quality monitoring systems. They formed a system with various sensors to detect the pH level, turbidity, conductivity of water and CO2, humidity, and temperature in the open air. The proposed model by Puja Sharma et al. [30] is intended to make the system cost-effective and affordable. So that anyone can freely use it. In their suggested system, information is obtained by multiple sensors, which are then transmitted to websites via the request form of the HTTP protocol to the website. But the lack of this method means that it focuses only on a few parameters, such as rain and temperature-related ones.

Monica J. et al. [31] suggested a weather monitoring system based on Internet of Things (IoT) technology to be built with the Proteus simulation tool. It collects all the required environmental information employing a sensor for temperature (LM35), a sensor for pressure (BMP180), a sensor to detect soil moisture, a rainfall detector, and a Raspberry Pi computer (RPI3) and then transmits that data directly into the ThingSpeak platform in order to predict the optimal conditions that are necessary for the growth of crops and yield enhancement. The IoT-based RTWMS created by A. Naveena et al. [32] allows for continuous monitoring of weather parameters involving the temperature, atmospheric pressure, humidity, rainfall, wind speed and gases including LPG, CO, and smoke. Through the ESP32's integrated Wi-Fi module, the sensor data is transmitted to the ThingSpeak cloud platform. Utilizing MATLAB analysis tools, this data is examined. In MATLAB, this data is read. On the ThingSpeak platform, the analysis is presented as a graph. The real-time weather monitoring system that was created in this way successfully monitors data and gives consumers trustworthy data, together with its graphical representation, all over the world. L.B. Imran et al. [33] proposed a Node MCU based temperature, turbidity, pH, dissolved oxygen findings in the smart city. Sensed data has been communicated and stored in LoRa cloud platform. H. Fuentes et al. [34] proposed a NodeMCU ESP8266 and Raspberry Pi based automatic smart water measurement and leakage finding system in smart cities. IBM cloud platform called NoSQL (Cloudant) used to display the information on webpage.

Sree Harini T et al. [35] proposed an intelligent environment monitoring system using IoT to get the information from regularly used weather variables and ThingSpeak cloud platform used to publish the information. Sudan Jha et al. [36] proposed smart city monitoring using IoT to find the air, noise, light and waste related atmospheric parameters in smart cities and also connected to GIS and solar. T.M. Bandara et al. [37] proposed an IoT based smart farm monitoring to protect the crop fields. Abdullah I et al. [38] proposed an IoT based smart waste water management, named “IoT-WMS” in Urban areas with five layers of blockchain technology which helped to reuse the waste water properly. P. Rathod et al. [39] developed a container monitoring system using IoT to safe the logistics using ATMega 328 and Node MCU processors along with DHT11 sensor, Smoke sensor and Magentic. Adafruit IO Cloud using the MQTT platform used to publish the data. The summary of the literature survey of the present experiment is illustrated in Table 1.

**Table 1:** Summary of the literature survey.

Author	Processing/Controlling Module Used	Parameter Measured	Cloud Platform Used
Kishore et al. [16]	Arduino Uno, ESP8266 Wi-Fi, NMCU, GSM.	Temperature, Humidity, Pressure Light, Raindrop	IBM Bluemix
Saini et al. [17]	Arduino	Temperature, Humidity, Pressure, Wind speed & Wind direction	GUI developed by LabVIEW 2012
Palle et al. [18]	CC3200 Launchpad	Humidity, Temperature	AT&TM2X Cloud Technology
Shaout et al. [19]	HCS12-MC9S12DG256	Wind speed, Wind direction, Temperature	–
Amber Katyal et al. [20]	ATmega328P MCU	Temperature, Humidity, Raindrop, Pressure	Thingspeak
Abu Saleh Bin Shahadat et al. [21]	ESP8266	Temperature, Pressure, Humidity, Rainfall.	Blynk – IoT
Shubham R. V. et al. [22]	ULN2803, Raspberry Pi	Humidity and temperature, pressure, altitude, light intensity and rain water level	–
Mohit Tiwari et al. [23]	ATMega 328 controller	temperature, humidity, and CO valu	IDE
R. Kavin et al. [24]	ATMega328, Wi-Fi	Light, Temperature, Humidity and Light	HTTP
Pasika and Gandla [25]	ATMega2560, ESP8266 Wi-Fi,	pH value, Turbidity, Water level, Temperature, Humidity	ThingSpeak
Kumar et al. [26]	Raspberry Pi	Water level, Raindrop, Temperature, Turbidity, pH level, MQ2 - CO_2_.	UBIDOTS
Gupta et al. [27]	ATMega328, ESP32-CAM	Turbidity, pH, Temperature	–
Vasanth K. Babu et al. [28]	Raspberry Pi-3, PIC MCU	Temperature, Humidity, Pressure, MQ2, MQ7, MQ135, MG811, Wind speed, Wind direction, Raindrop, Water level	HTTP
Varsha et al. [29]	ATMega328	Temperature, Humidity, pH, Conductivity, Turbidity	–
Puja Sharma et al. [30]	Node MCU, Wi-Fi	Temperature, Pressure, Raindrop	HTTP
Monica J. et al. [31]	Raspberry Pi (RPI3),	Temperature, Pressure, Raindrop, Soil Moisture	ThingSpeak
A. Naveena et al. [32]	ESP32 Wi-Fi	Temperature, Pressure, Raindrop, Gas	ThingSpeak
L.B. Imran et al. [33]	Node MCU	Temperature, Turbidity, pH, Oxygen.	LoRa
H. Fuentes et al. [34]	NodeMCU ESP8266, Raspberry Pi, Smart water meter	Water leakage	NoSQL (Cloudant)
Sree Harini T et al. [35]	Arduino Uno, ESP8266	Temperature, Humidity, Light and Pressure sensors, LCD	ThingSpeak
Sudan Jha et al. [36]	MCU, Webcam, Smart water bin	Temperature, humidity, smoke sound, motion, fire, GIS and solar	–
T.M. Bandara et al. [37]	Smart Devices, Sensor Node	Soil moisture, water volume, temperature	–
Abdullah I et al. [38]	IoT-WMS	WSN	Cloud Security using Blockchain Technology
P. Rathod et al. [39]	ATMega 328 Node MCU	Temperature, Humidity, Smoke	Adafruit IO Cloud using the MQTT

According to the literature, just a few of the sensors were used. In the present article, we provided an enhanced real-time smart monitoring system using IoT and sensors to address the issues identified in the preceding research studies. The system enables individuals to remotely observe the surroundings. The architecture includes a graphical user interface which enables people to not only observe the information but also control the system. The subsequent steps define the comprehensive methodology.

- It concentrated on all of the important atmospheric characteristics associated with air, water, waste, noise, and energy;
- The essential factors we concentrated on here are the humidity, temperature, pressure in the environment, the speed and direction of the wind, the quality of the air, hazardous gases, rainfall, water level, the value of pH, moisture content of the soil, degree of light, and turbidity;
- It will help to monitor the weather conditions and their changes individually without depending on the forecasting agencies, detect the hazardous gases and air quality and alert the mining people detect the water quality, alert the farmers in risky weather conditions, enhance the wildlife ness of animals and people, alert urban citizens, alter the people from floods, avoid the accidents in hazardous places, used to supply pure drinking water, give information by monitoring the weather conditions and by analyzing the changes in the near climate to know and increase the necessity environmental safety and protection, create the awareness and enhance the utilization of real-time smart monitoring system for uneducated people to know the environmental parameters changes and its consequences what will happen in future;
- Sensor nodes are devices that periodically assess their surroundings.
- Data is acquired by sensors at predetermined time intervals determined by sensing duration;
- Data is sent to a gateway device, which filters it for the IoT server;
- The gateway device then uploads data to the IoT cloud for storage and analysis;
- This information is visualized for the system's user;
- Cloud-based server algorithms analyze information gathered by sensor nodes and alter the threshold and time frame for monitoring.

The proposed research article is structured as follows: Section 1 gives the introduction about the environmental parameters and possibilities of smart monitoring of them. Also provides the literature survey related to the projects, research findings and organization of the present article. Section 2 describes the methods and materials such as hardware & software components and schematic diagram. Section 3 presents the results and discussion. At final section 4 gives conclusion and future scope of the project.

## 2 Materials and methods

The proposed system is designed to monitor and measure various environmental parameters such as temperature, humidity, atmospheric pressure, hazardous gases, air quality, raindrops (rainfall detection), rain gauges (flood alert and water level measurement), water quality such as pH and turbidity, light intensity, soil moisture, wind speed, and direction.

**Fig. 1:**
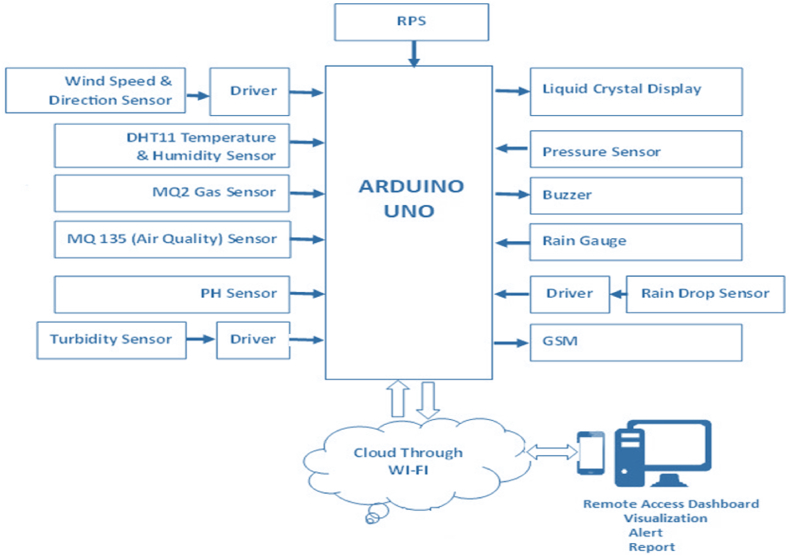
Block diagram of a smart environmental parameter monitoring system.

The main goal of our design is to demonstrate sophisticated real-time smart monitoring of environmental factors based on IoT and sensor networks that keeps track of weather conditions independently of forecasting agencies. By placing sensor modules in the surrounding area and analyzing real-time data gathered and monitored on a webpage by utilizing a Wi-Fi-based HTTP IoT platform, the system's performance is made possible [40]. Arduino Uno and other diverse sensors are used in the hardware configuration to collect field data and manage various environmental-related operations. The hardware and software used for the experiment are illustrated in Fig. 1.

The prototype produced in this experiment monitors several meteorological factors in the atmosphere, such as temperature, pressure, gas, smoke, air quality, humidity, wind speed and direction, rainfall, rain level, pH value, and turbidity [41]. There are four operational layers in the proposed concept. The first one is the sensor deployment layer, which is used to sense the data from the atmosphere. The second is the middleware layer, which sends the collected data from sensors to a microcontroller based on the Arduino Uno that supervises the whole process. The third one is the communication layer, which is in control of interfacing with all of the installed sensors from the microcontroller to the output modules. That collected data is transferred to the output modules called buzzer, LCD, and GSM. The fourth is the cloud analysis layer, or application layer, which is responsible for a Wi-Fi-enabled IoT-based HTTP webpage to communicate the information wirelessly to a remote user to report and alert.

IoT-based monitoring systems rely on microcontroller local storage for offline storage and quick data access. Real-time data transmission entails transferring sensor data to a central server or cloud for control and scaling. The Arduino Uno microcontroller is suited for small-scale, but not frequent, storage. SD card modules and serial connectivity are both options for additional storage.

### 2.1 Hardware components

The aforementioned components for data collection, data transfer, data processing and analysis are employed in developing of the proposed IoT-based wireless smart monitoring system for parameters associated with the environment. For smart monitoring of environmental parameters different sensors are used in this work. The sensors are selected based on the numerous significant variables like accuracy, calibration, power consumption, compatibility, atmospheric condition, dependability, durability, and cost. After extensively investigated and analyzed different sensor solutions based on these requirements the effective sensors are used for environmental monitoring.

#### 2.1.1 Arduino Uno (ATMega1280)

A microcontroller board based on the ATMega1280 is called the Arduino Mega. It has 16 analogue inputs, 4 physical serial connectors (UARTs), a crystal oscillator with a bandwidth of 16 MHz, 54 logical input/output data transfer pins (14 of which serve as PWM signals, a USB interface, an external power connector, an ICSP header for communication, along with a reset switch. Another Type-B USB interface for supplying and coding a microcontroller is located on the board's left short edge. It serves as an open-source and free platform for electronic development that comprises adaptive and intuitive both software and hardware. It has several features, such as operating on a 5V power source and operating between 5V and 20V, 28 KB of flash memory, 4 KB of which is required for the boot loader, 8 KB of SRAM, and 4 KB of EEPROM. The ATMega 2560 has same features but only difference is it offers additional 128 KB of flash memory [42].

#### 2.1.2 Temperature & humidity sensor (DHT11)

An effortless, inexpensive digital temperature cum humidity detector is the DHT11 which records temperature and humidity data at regular intervals depending on the adjusted digital signal output. It has a 5% accuracy over a humidity range of 20–80% and a temperature range of 0–50 °C. To determine the current temperature, A thermistor and a capacitive humidity sensor are both used by the DHT11. It runs at 5 V with a maximum current of 2.5 mA [43].

#### 2.1.3 Pressure sensor (BMP280)

BMP280 is a piezo-resistive digital pressure sensor that measures barometric pressure, temperature humidity and altitude. It supports both SPI and I2C communication interfaces. This system has been used to measure surrounding atmospheric pressure using a serial I2C interface directly to a microcontroller [44].

#### 2.1.4 Gas sensor (MQ2)

Chemiresistors are another name for MQ2 gas sensors. When the gas makes contact with the sensor material, the resistance changes, which is detected and converted to an analogue form. The gas content is determined using a voltage divider structure. It operates at 5V DC and consumes approximately 800 mW of power. The exhaust via the sensing device gets transformed into a voltage that is an appropriate representation (i.e., PPM). The design measures natural gas (LPG smoke, beverages, transportation fuel, and greenhouse gases such as methane, hydrogen, and CO) across an identification spectrum that includes 200 to 10,000 PPM [45,46].

#### 2.1.5 Air quality sensor (MQ135)

The MQ135 serves as a gaseous or alcoholic analyzer, which examines the condition of the air around it. The oxide of tin (SnO2) is the most significant constituent. It recognizes ammonia, sulfur dioxide, and benzine moisture, as well as pollutants and various other poisonous gases. The device is economical and can be employed for an assortment of objectives, including detecting hazardous gases and smoke. It is useful for NH3, nitrogen oxides (NO), and other smoke findings. It identifies gasoline and alcohol in a form of electricity that is voltage (i.e., PPM) [47].

#### 2.1.6 Rain drop sensor

Raindrops were successfully detected by raindrop sensors. The design comprises a pair of autonomous modules: a rain panel to monitor rainfall and a control module for analyzing and contrasting conventional and digital measurements. It is a simple and inexpensive device that assists in sensing rainfall and functions at 5 V by triggering a switch when drops descend on the sensor panel [48].

#### 2.1.7 Rain gauge (ultrasonic sensor)

This sensor is set at the leading edge of a container and directed downward. It emits waves and counts the amount of time that it requires to get the response signal from the water to reach the sensor for the purpose to estimate the levels of any kind of liquid or solid. This sensor operates by emitting high-frequency sonic waves at regular time intervals, and the quantity of returned sound waves is proportional to the distance between the transducer and the object to be determined. For example, water range information is provided from 6 inches to 254 inches with a measurement accuracy of 1 inch, and the informational sheet for this particular item indicates that the surface of the ocean object covers a distance of 0 inches–254 inches (6.54 m) [49].

#### 2.1.8 pH sensor

It uses three separate that are soil, liquid and light test meters in one gadget to correctly and efficiently assess pH/acidity level, moisture content, and light amount. It does not require a battery and can be readily plugged into the soil to detect results automatically. Compact and portable, it is able to be used in lawns, gardens, potted plants, industrial settings, and many other places [50].

#### 2.1.9 Turbidity sensor (SEN0189)

Through the measurement of turbidity, water quality is determined. By detecting light transmittance and producing signals in either analogue or digital formats, depending on the microcontroller unit (MCU), the turbidity sensor makes it possible to identify suspended particles in water. Nephelometric turbidity units (NTU) are used to measure water quality; less than 1 NTU is considered good, 1 to 5 NTU is considered fair, and more than 5 NTU is considered poor [51].

#### 2.1.10 Wind speed and direction sensor (DC motor with propeller)

A device used to measure the direction and speed of the wind is an anemometer. It is a typical piece of weather station equipment. The wind anemometer in our suggested system was a DC motor. The flux change that causes the generation of EMF on an ATMega microcontroller is measured and proportionate wind speed is calculated. The direction of the wind is measured using digital compass-based equipment [52].

#### 2.1.11 ESP8688 Wi-Fi module

The hardware is dependent on the ESP12E Module, and Node MCU has firmware that is compatible with and works on the ESP8266 Wi-Fi System on Chip (SoC). The ESP8266 is inexpensive, includes an in-built Wi-Fi module, and uses ultra-low-power technologies [53].

#### 2.1.12 Buzzer

A buzzer is an auditory signaling device that can be mechanical, electromechanical, or both (piezoelectric). It is used to sound an alert when the parameter value reaches a particular threshold.

#### 2.1.13 LCD

The capacity of liquid crystals to control light is used to produce the text or image on a flat-panel electronic visual display known as a liquid-crystal display (LCD). An alphanumeric monochrome 20x4 LCD was employed. The 20x4 format enables the simultaneous display of 20 characters in each of the 4 rows of the 20x4 LCD, for a total of 80 characters.

#### 2.1.14 GSM

GSM - The GSM module is a dual-mode information transfer module. The main parts of a GSM module are the GSM baseband CPU, flash, GSM Radio Frequency (RF), power, antennas and their jacks. SIMCOM manufactures the module, which has an operational voltage range of 3.2V–4.8V and low power consumption. It supports the 900 MHZ and 1800 MHZ frequency bands. Data and voice are transported safely and quickly within the frequency spectrum. Two different SMS modalities are supported by the GSM module called a text mode and a PDU mode respectively [54]. Due to technology's ability to deliver digital communications, we selected text mode.

#### 2.1.15 Communication devices and protocols

To transfer the data from the microcontroller to other peripherals the communication protocols are commonly used. In this work SPI (Serial Peripheral Interface) is used for high-speed communication from microcontrollers to other peripherals like sensors, memory chips, and displays. The USB (Universal Serial Bus) is used for both power and data transfer. It supports various data transfer speeds and device classes. Finally, the wireless protocols such as Wi-Fi are used to access the data through internet.

### 2.2 Schematic diagram

**Fig. 2:**
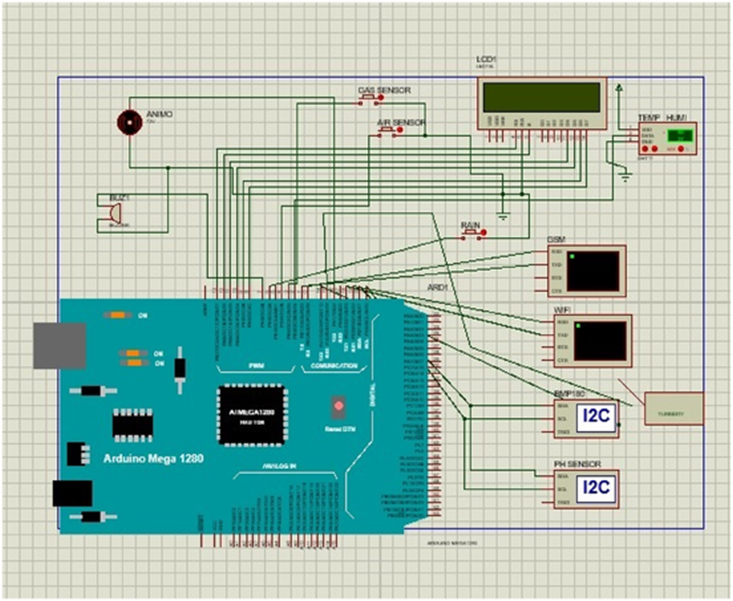
Schematic diagram of a smart monitoring system for measuring environmental parameters.

Various components are interfaced in the design of the proposed system to develop a smart system for monitoring weather parameters. interfaced Table 2 lists the components provided with diagrams. As a result of the connecting of the components, a schematic diagram has been shaped. The schematic diagram of the hardware components and their connections is shown in Fig. 2.

**Table 2:**
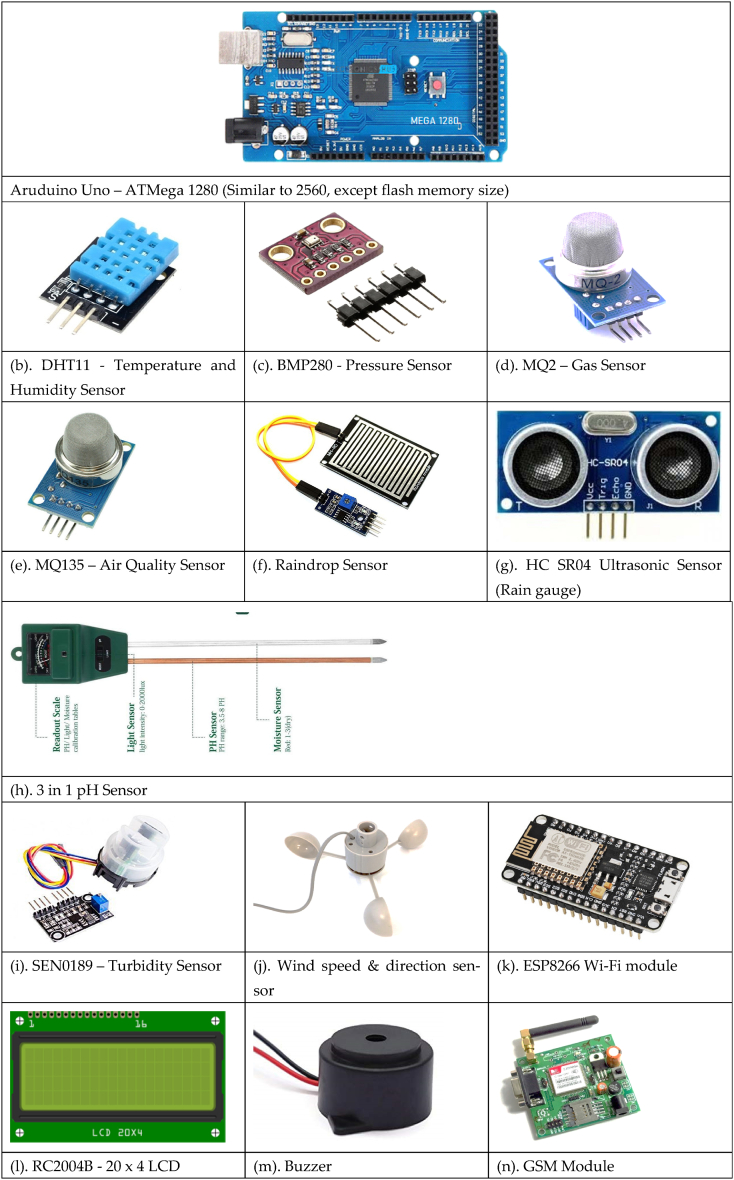
List of the hardware used components used develop the proposed system.

### 2.3 Software requirements

Each piece of hardware must be properly configured in accordance with the schematic diagram and programmed to work with the other pieces of hardware in order for the environmental parameter monitoring system to work. The Arduino microcontroller, which also contains the code to direct and manage the operation of the other gear, acts as the system's processor. In this proposed work the Arduino IDE 1.8.13 version is used because it is widely used for writing computer programs that can be transferred directly to a hardware board. Also in this work, the libraries like Wire (Version 1.0.1), SPI (Version 1.0.1), Adafruit GFX (Version 1.10.0), Adafruit Motor Shield (Version 1.0.2) were utilized. The entire monitoring system was written in C, with assembly language incorporated [55]. The programming environment of the Arduino IDE includes a number of useful sample codes that can be expanded to carry out more complex functions through connected libraries which created an HTTP-based webpage to remotely monitor and examine the data.

The user interface on an HTTP-based webpage for an environmental monitoring system should prioritize user friendliness and accessibility to ensure a smooth and intuitive experience for users. It can be considered in various aspects, such as clear and intuitive navigation, understanding and interacting with the system to access and analyze the collected data effectively, responsive design, visual presentation, dashboard overview, interactive controls, data visualization and analysis, alerts and notifications, and accessibility considerations.

Implementing security measures is critical for safeguarding data transmission and ensuring the system's resilience to potential cyber assaults. Transport Layer Security (TLS) ensures secure communication between the user's browser and the server that hosts the website. It encrypts data sent across the network to prevent unauthorized access or interception.

### 2.4 Power requirements (regulated power supply)

The power supply method of a system has a substantial impact on its energy efficiency, power management, power storage, and sustainability. These are the factors for building a power supply framework that supports energy efficiency and sustainability while lowering the system's environmental impact and ensuring long-term survival. The power supplies are intended to convert high-voltage AC mains electricity into a suitable low-voltage supply for electronic circuits and other devices. A power supply can be broken down into a series of blocks, each of which serves a specific purpose. The term "regulated DC power supply" refers to a DC power source that keeps the output voltage constant despite fluctuations in the AC mains or load. Batteries and a solar panel with an inverter were used as a backup power supply mechanism.

### 2.5 Ethical considerations

Ethical considerations play a crucial role in environmental monitoring, particularly when data collection involves sensitive areas. Ethical considerations should be an integral part of the planning, implementation, and ongoing management of environmental monitoring projects. When conducting environmental monitoring in sensitive areas, it is important to obtain informed consent from individuals or communities affected by the monitoring activities. This ensures that they are aware of the purpose, potential risks, and benefits of the monitoring project. Adhering to ethical principles ensures that monitoring activities are conducted in a responsible, respectful, and data-protective manner. Transparency builds trust and allows for public scrutiny, enabling stakeholders to understand and engage with the data collection process in an accountable manner, considering any potential ecological or social risks, fostering trust, and promoting the well-being of both the environment and the communities involved.

### 2.6 Societal impacts

The proposed real-time smart environmental parameter monitoring technology using IoT and sensors has the potential to create significant societal impacts, particularly in terms of environmental conservation and safety, by enabling continuous and accurate data collection of environmental parameters such as air quality, water quality, noise levels, and GSM, TFT, W-Fi, and other libraries more. This data can provide valuable insights into the health and condition of ecosystems, helping in the identification of environmental issues, pollution sources, and ecological changes. With this information, timely and targeted interventions can be implemented to mitigate environmental degradation, protect biodiversity, and promote sustainable resource management. This system served as an early warning system for detecting and alerting to potential environmental hazards or safety concerns, data-driven decision-making, public awareness and engagement, and improved resource efficiency. These advancements have the potential to create a more sustainable and resilient future where the well-being of both the environment and the communities that depend on it is safeguarded.

## 3 Results and discussion

The experiment is conducted at ‘KLM College of Engineering form Women’ and its surroundings, Kadapa, Andhra Pradesh, India from April to July 2023. When the RPS is turned on by connecting a pin to the appropriate slot, all the sensors that are pressure sensor, humidity & ambient temperature sensor, wind speed and direction sensor, raindrop sensor, rain gauge sensor, pH sensor, MQ2 gas sensor, MQ135 air quality sensor, and turbidity sensor are initialized and comes under the process of sensing the environmental parameters data, LCD displays a welcome note on its screen. GSM which we had previously installed into the GSM module slot is automatically initialized by sending a text message to the registered mobile number from another GSM number which we want to receive the collected data.

Sensor data collection and storage-based timeframes depend on factors like application, sensor type, and desired analysis level. Real-time monitoring requires short intervals, while long-term trend analysis may require longer ones. So, In this work here the data from sensors are collected for different time slots like every minute and every 10 min regularly at ‘KLM College of Engineering for Women’ and its surroundings, Kadapa, Andhra Pradesh, India, from April to July 2023, and provided meaningful analysis.

Initialized the Wi-Fi by turn on the Wi-Fi connection and keep it active until the relevant data is obtained. So once the initialization of all the components has been completed then the sensors started continuously sensing the environmental conditions by respective sensors. All the components are working under the supremacy of ATMega 1280 MCU. The sensed data from sensors are fetch onto the LCD. The same data simultaneously send from the GSM to registered mobile number, Wi-Fi also communicate simultaneously and automatically sent data same sensed environmental information to display on the website by HTTP-based protocol. The proposed system will sound a warning through a buzzer if it detects changes in the environmental parameters above the threshold values.

**Fig. 3:**
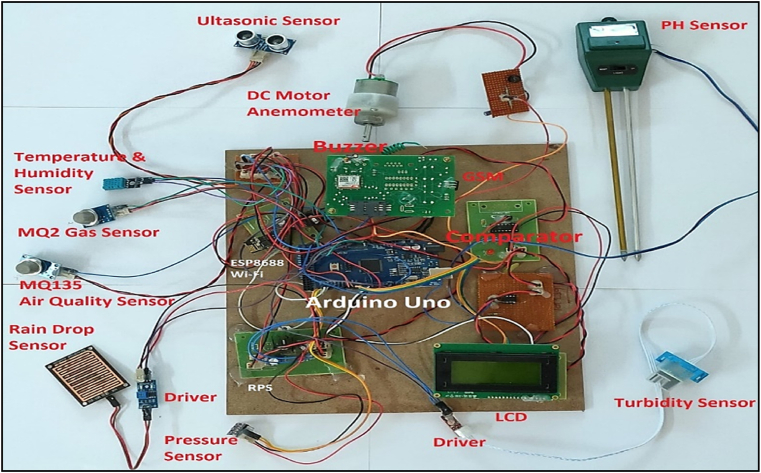
Experimental setup of a smart monitoring system for environmental parameters.

**Fig. 4:**
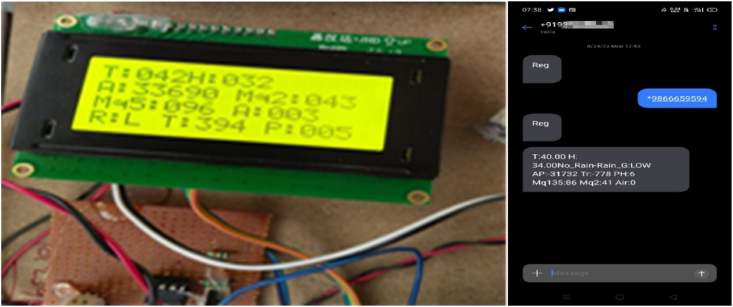
Output display of the collected data on the LCD and on the GSM (received SMS).

**Fig. 5:**
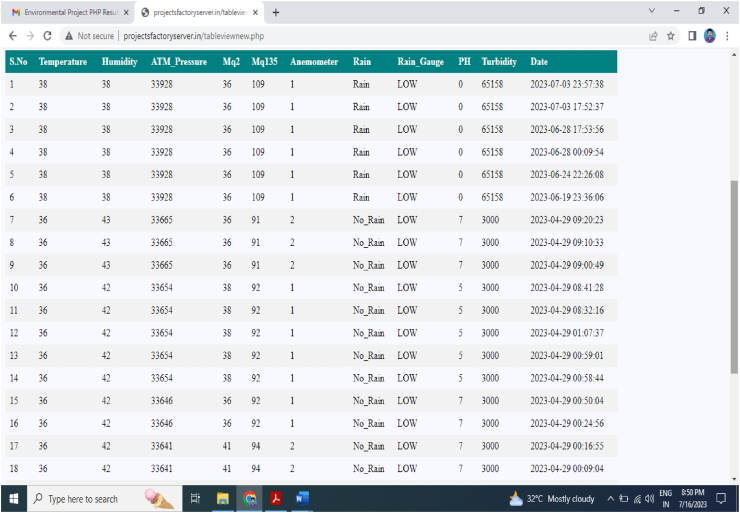
Display of obtained data's real-time parameters on a webpage.

**Fig. 6:**
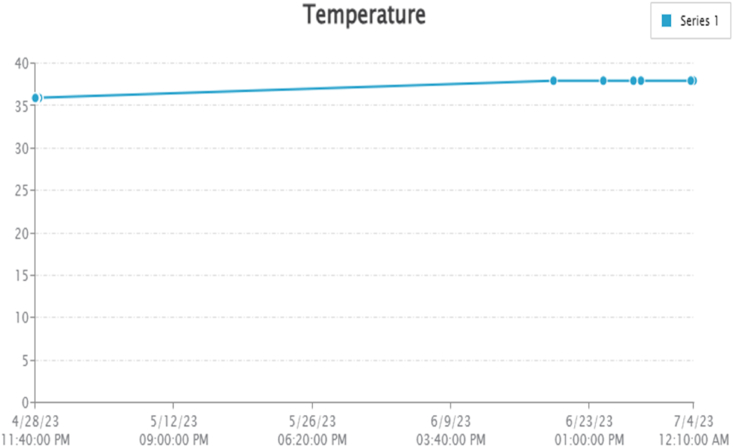
Graphical representations of the experimental findings of temperature sensor.

**Fig. 7:**
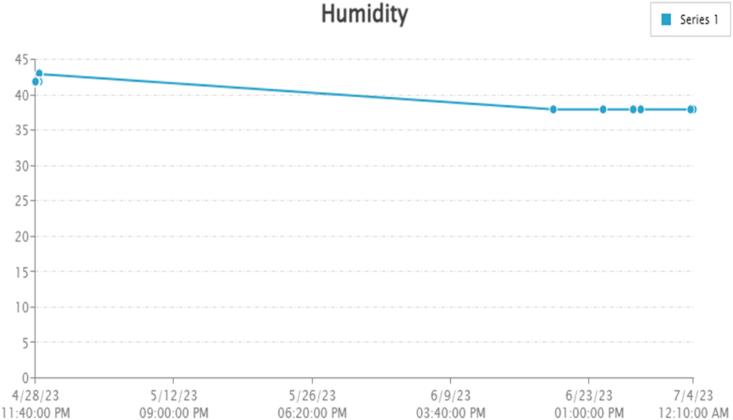
Graphical representations of the experimental findings of humidity sensor.

**Fig. 8:**
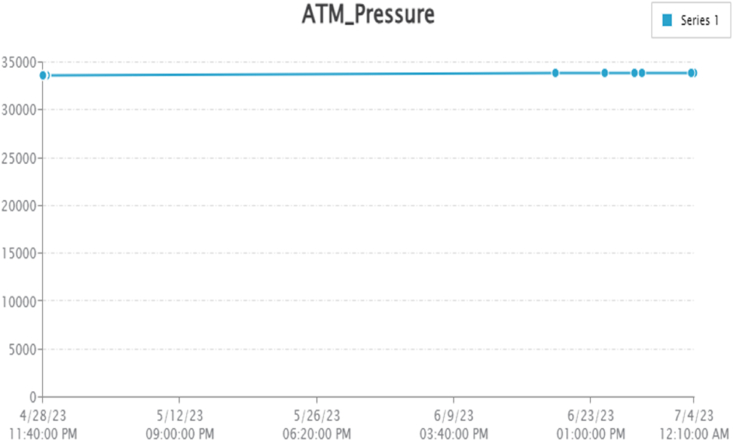
Graphical representations of the experimental finding of atmospheric pressure sensor.

**Fig. 9:**
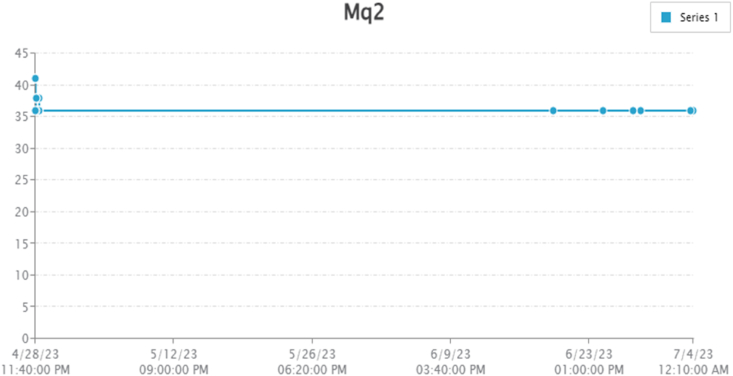
Graphical representations of the experimental findings of gas temperature sensor.

**Fig. 10:**
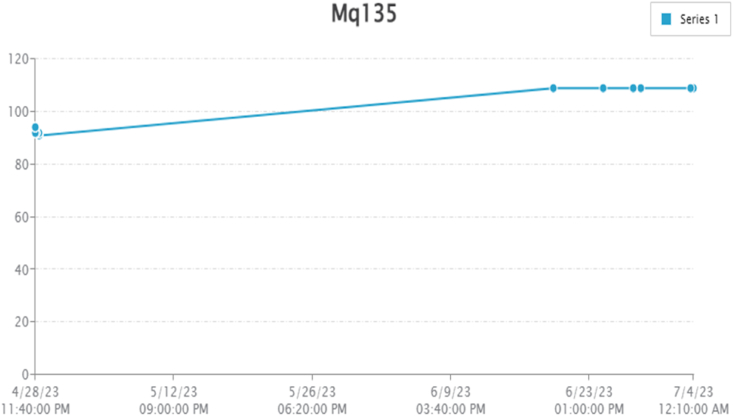
Graphical representations of the experimental findings of air quality sensor.

**Fig. 11:**
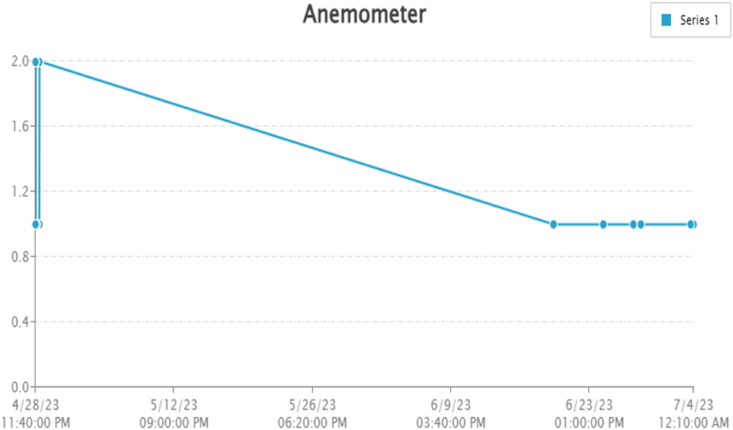
Graphical representations of the experimental findings of anemometer.

**Fig. 12:**
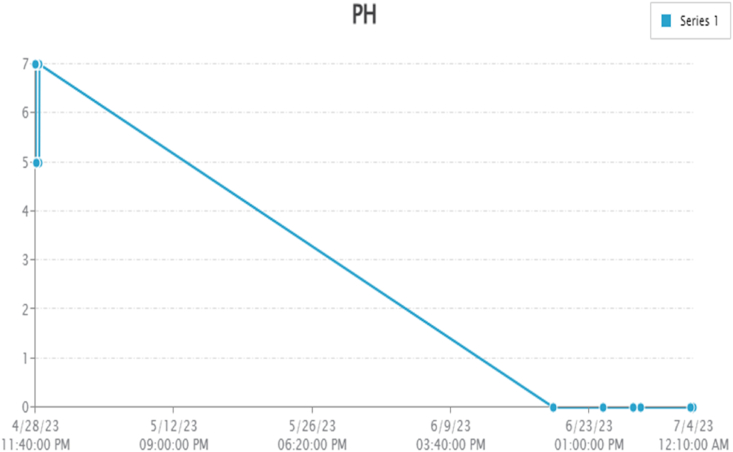
Graphical representations of the experimental findings by pH sensor.

**Fig. 13:**
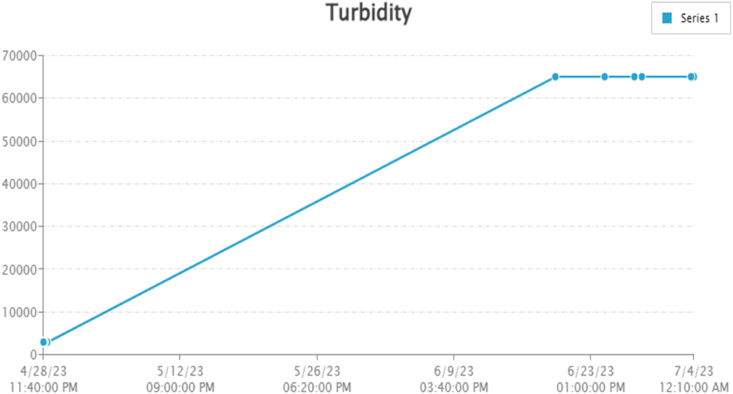
Graphical representations of the experimental findings of turbidity sensor.

By taking account of all the processes it is clear that the proposed system has been developed with the goal of multiple offers of communication for any individual to monitor the atmospheric changes remotely. Those are 1). LCD for direct monitoring, 2). Buzzer to alert when threshold level reached, 3). GSM to monitor by receiving the SMS when the people at distance from the system and 4) Webpage to monitor even at far long distance. Here, the visual and digital findings of the experiment are available. Fig. 3 illustrates the experimental setup of a smart monitoring system for environmental parameters. Fig. 4 illustrates the environmental findings of various parameters by various wireless sensors, including a pressure sensor, a humidity & ambient temperature sensor, a pressure sensor, a wind speed and direction sensor, a raindrop sensor, a rain gauge sensor, a pH sensor, an MQ2 gas sensor, an MQ135 air quality sensor, and a turbidity sensor on an LCD and GSM. To inform the remote user and control the appropriate atmosphere to prevent dangers, the detected reading of the proposed experimentation is also presented on the webpage using the Internet of Things. Results from wireless sensors are shown on an HTTP-enabled webpage which is illustrated in Fig. 5. The real time environmental parameters obtained at KLM College of Engineering for Women, Kadapa are tabulated in tables from Table 3, Table 4, Table 5, Table 6, Table 7, Table 8, Table 9, Table 10, and their graphical representations shown in Fig. 6, Fig. 7, Fig. 8, Fig. 9, Fig. 10, Fig. 11, Fig. 12, Fig. 13 are generated automatically by the software code which is developed to interface the hardware components used in the proposed system and their respectively. Based on the above results, it is feasible to send alerts to those who need to be aware and professional depending on the atmospheric conditions. Table 11 gives the indications or alerts based on observing and analyzing the data of the observers. Note that the SI units for all measured parameters are as follows: Temperature in degrees Celsius (°C), humidity in percentages (%), pressure in hectopascals (hPa), Gas concentration in parts per million (ppm), Air quality index (no specific unit), Raindrop or rain intensity (no specific unit), Rainfall in millimeters (mm), pH value (no unit), Turbidity in Nephelometric Turbidity Units (NTU), kilometers per hour (km/h), Direction in degrees (°) or cardinal directions (e.g., north, south, etc.)

**Table 3:** Obtained experimental findings by temperature sensor.

S. No	Temperature	
	Date and Time	Degree Centigrade (^o^C)
1	2023-04-28 23:42:24	36
2	2023-04-29 00:00:36	36
3	2023-04-29 00:09:04	36
4	2023-04-29 00:16:55	36
5	2023-04-29 00:24:56	36
6	2023-04-29 00:50:04	36
7	2023-04-29 00:58:44	36
8	2023-04-29 00:59:01	36
9	2023-04-29 01:07:37	36
10	2023-04-29 08:32:16	36
11	2023-04-29 08:41:28	36
12	2023-04-29 09:00:49	36
13	2023-04-29 09:10:33	36
14	2023-04-29 09:20:23	36
15	2023-06-19 23:36:06	38
16	2023-06-24 22:26:08	38
17	2023-06-28 00:09:54	38
18	2023-06-28 17:53:56	38
19	2023-07-03 17:52:37	38
20	2023-07-03 23:57:38	38

**Table 4:** Obtained experimental findings by humidity sensor.

S. No	Humidity	
	Date and Time	g/kg (%)
1	2023-04-28 23:42:24	42
2	2023-04-29 00:00:36	42
3	2023-04-29 00:09:04	42
4	2023-04-29 00:16:55	42
5	2023-04-29 00:24:56	42
6	2023-04-29 00:50:04	42
7	2023-04-29 00:58:44	42
8	2023-04-29 00:59:01	42
9	2023-04-29 01:07:37	42
10	2023-04-29 08:32:16	42
11	2023-04-29 08:41:28	42
12	2023-04-29 09:00:49	43
13	2023-04-29 09:10:33	43
14	2023-04-29 09:20:23	43
15	2023-06-19 23:36:06	38
16	2023-06-24 22:26:08	38
17	2023-06-28 00:09:54	38
18	2023-06-28 17:53:56	38
19	2023-07-03 17:52:37	38
20	2023-07-03 23:57:38	38

**Table 5:** Obtained experimental findings by pressure sensor.

S. No	ATM Pressure	
	Date and Time	hPa = 1000*pa
1	2023-04-28 23:42:24	33,641
2	2023-04-29 00:00:36	33,641
3	2023-04-29 00:09:04	33,641
4	2023-04-29 00:16:55	33,641
5	2023-04-29 00:24:56	33,646
6	2023-04-29 00:50:04	33,646
7	2023-04-29 00:58:44	33,654
8	2023-04-29 00:59:01	33,654
9	2023-04-29 01:07:37	33,654
10	2023-04-29 08:32:16	33,654
11	2023-04-29 08:41:28	33,654
12	2023-04-29 09:00:49	33,665
13	2023-04-29 09:10:33	33,665
14	2023-04-29 09:20:23	33,665
15	2023-06-19 23:36:06	33,928
16	2023-06-24 22:26:08	33,928
17	2023-06-28 00:09:54	33,928
18	2023-06-28 17:53:56	33,928
19	2023-07-03 17:52:37	33,928
20	2023-07-03 23:57:38	33,928

**Table 6:** Obtained experimental findings by gas sensor.

S. No	Mq2	
	Date and Time	Part per million (ppm)
1	2023-04-28 23:42:24	41
2	2023-04-29 00:00:36	41
3	2023-04-29 00:09:04	41
4	2023-04-29 00:16:55	41
5	2023-04-29 00:24:56	36
6	2023-04-29 00:50:04	36
7	2023-04-29 00:58:44	38
8	2023-04-29 00:59:01	38
9	2023-04-29 01:07:37	38
10	2023-04-29 08:32:16	38
11	2023-04-29 08:41:28	38
12	2023-04-29 09:00:49	36
13	2023-04-29 09:10:33	36
14	2023-04-29 09:20:23	36
15	2023-06-19 23:36:06	36
16	2023-06-24 22:26:08	36
17	2023-06-28 00:09:54	36
18	2023-06-28 17:53:56	36
19	2023-07-03 17:52:37	36
20	2023-07-03 23:57:38	36

**Table 7:** Obtained experimental findings by air quality sensor.

S. No	Mq135	
	Date and Time	Part per million (ppm)
1	2023-04-28 23:42:24	94
2	2023-04-29 00:00:36	94
3	2023-04-29 00:09:04	94
4	2023-04-29 00:16:55	94
5	2023-04-29 00:24:56	92
6	2023-04-29 00:50:04	92
7	2023-04-29 00:58:44	92
8	2023-04-29 00:59:01	92
9	2023-04-29 01:07:37	92
10	2023-04-29 08:32:16	92
11	2023-04-29 08:41:28	92
12	2023-04-29 09:00:49	91
13	2023-04-29 09:10:33	91
14	2023-04-29 09:20:23	91
15	2023-06-19 23:36:06	109
16	2023-06-24 22:26:08	109
17	2023-06-28 00:09:54	109
18	2023-06-28 17:53:56	109
19	2023-07-03 17:52:37	109
20	2023-07-03 23:57:38	109

**Table 8:** Obtained experimental findings by anemometer.

S. No	Anemometer	
	Date and Time	Propeller Direction (1-Clockwise, 2 Counter Clock)
1	2023-04-28 23:42:24	2
2	2023-04-29 00:00:36	2
3	2023-04-29 00:09:04	2
4	2023-04-29 00:16:55	2
5	2023-04-29 00:24:56	1
6	2023-04-29 00:50:04	1
7	2023-04-29 00:58:44	1
8	2023-04-29 00:59:01	1
9	2023-04-29 01:07:37	1
10	2023-04-29 08:32:16	1
11	2023-04-29 08:41:28	1
12	2023-04-29 09:00:49	2
13	2023-04-29 09:10:33	2
14	2023-04-29 09:20:23	2
15	2023-06-19 23:36:06	1
16	2023-06-24 22:26:08	1
17	2023-06-28 00:09:54	1
18	2023-06-28 17:53:56	1
19	2023-07-03 17:52:37	1
20	2023-07-03 23:57:38	1

**Table 9:** Obtained experimental findings by pH sensor.

• **S. No**	•PH	
	**Date and Time**	**PH Scale (0-13)**
1	2023-04-28 23:42:24	7
2	2023-04-29 00:00:36	7
3	2023-04-29 00:09:04	7
4	2023-04-29 00:16:55	7
5	2023-04-29 00:24:56	7
6	2023-04-29 00:50:04	7
7	2023-04-29 00:58:44	5
8	2023-04-29 00:59:01	5
9	2023-04-29 01:07:37	5
10	2023-04-29 08:32:16	5
11	2023-04-29 08:41:28	5
12	2023-04-29 09:00:49	7
13	2023-04-29 09:10:33	7
14	2023-04-29 09:20:23	7
15	2023-06-19 23:36:06	0
16	2023-06-24 22:26:08	0
17	2023-06-28 00:09:54	0
18	2023-06-28 17:53:56	0
19	2023-07-03 17:52:37	0
20	2023-07-03 23:57:38	0

**Table 10:** Obtained experimental findings by turbidity sensor.

S. No	Turbidity		
	Date and Time	Finding value	Nephelometric Turbidity Units (NTUs)
1	2023-04-28 23:42:24	3000	0.3
2	2023-04-29 00:00:36	3000	0.3
3	2023-04-29 00:09:04	3000	0.3
4	2023-04-29 00:16:55	3000	0.3
5	2023-04-29 00:24:56	3000	0.3
6	2023-04-29 00:50:04	3000	0.3
7	2023-04-29 00:58:44	3000	0.3
8	2023-04-29 00:59:01	3000	0.3
9	2023-04-29 01:07:37	3000	0.3
10	2023-04-29 08:32:16	3000	0.3
11	2023-04-29 08:41:28	3000	0.3
12	2023-04-29 09:00:49	3000	0.3
13	2023-04-29 09:10:33	3000	0.3
14	2023-04-29 09:20:23	3000	0.3
15	2023-06-19 23:36:06	65,158	6.5
16	2023-06-24 22:26:08	65,158	6.5
17	2023-06-28 00:09:54	65,158	6.5
18	2023-06-28 17:53:56	65,158	6.5
19	2023-07-03 17:52:37	65,158	6.5
20	2023-07-03 23:57:38	65,158	6.5

**Table 11:** Sensor output indicators for various circumstances.

S. No	Sensor	Indicator
1	DHT11 - Temp & Hum Sensor	The soil will dry out at high temperatures.
2	BMP280 - Pressure Sensor	A higher value indicates the pressure of the atmosphere.
3	MQ2 - Gas Sensor	A higher value implies that there is smoking in the surrounding territory.
4	MQ135 - Air Quality Sensor	Higher readings represent poor conditions for the air.
5	Raindrop Sensor	A higher number signifies that it is rainy.
6	Rain Gauge (Flood Alert Sensor)	The sensor's value represents the amount of rain (water level). Give the flood alerts also.
7	pH Sensor (Multipurpose)	Determines the alkali content of the water.
		Measure the moisture content of the atmosphere.
		Identify the light intensity level.
8	SEN0189 – Turbidity Sensor	A higher value indicates more pollution and turbidity in the water.
11	Wind Speed	Determine the most rapid airflow velocity.
12	Wind Direction	Give the direction of the wind.

### 3.1 Raindrop sensor

Raindrop sensor detects rain when any drop placed on the sensor. Fig. 5 shows ‘Rain’ when there is water drop on the sensor.

### 3.2 Rain gauge (ultrasonic sensor)

Since the ultrasonic sensor detects the digital value unambiguously, it is interpreted as the duration of time in seconds. Eq. (1) is used to determine distance with the help of the duration.

(1) Distance = (Duration) / 58.8

### 3.3 Temperature and humidity sensor

Temperature and humidity of the environment around KLMCEW, Kadapa are determined from the DHT-11 sensor module and updated in the HTTP based webpage, as illustrated in Fig. 5. The temperature is measured in degrees Celsius, while the humidity is expressed as a percentage with units measured in g/kg. Because the pH and turbidity sensors provide reliable values in specified air conditions, the temperature of the surrounding area is computed. As illustrated Table 3, Table 4 provided the measured values of temperature and humidity respectively. Similarly, Fig. 6, Fig. 7 offered the graphical representation of temperature and humidity of the surrounding atmosphere are additionally recorded in the serial monitor interface of the Arduino IDE with Wi-Fi and PHP webpage by HTTP. The maximum temperature computed at time our experiment is 38 °C and minimum temperature is 36 °C., maximum and minimum humidity percentage findings are 43 g/kg and 38 g/kg respectively.

### 3.4 Atmospheric pressure sensor

The test is carried out by comparing the data from the BMP280 Pressure sensor measurement results, which are displayed in Table 5. Fig. 8 depicts a graphical representation of a pressure sensor. The pressure values obtained a maximum of 33,928 hpa and minimum of 33,641 which are observed in Table 5.

### 3.5 Gas sensor

Gas sensor is used find the harmful gases in the atmosphere. The experimental finding of the gas identification is shown in Table 6 and the same readings are represented graphically as shown in Fig. 9.

### 3.6 Air quality sensor

Air quality by using MQ135 sensor is used find the air pollution in the atmosphere. The air quality is measured with the unit “ppm” means part per million. The minimum and maximum values of air quality are 91 ppm and 94 ppm respectively. Experimentally obtained reading are placed in Table 7 and same are graphically represented in Fig. 10.

### 3.7 Anemometer (wind speed and direction)

General DC motor with propeller is used here as anemometer to find the wind direction. Here, the reading 1 represents the clockwise direction and reading 2 represents counter clockwise direction. Table 8 and Fig. 11 gives the wind direction. Note the propeller placed in east face direction.

### 3.8 pH sensor

Every 20 s, the computer that hosts the server has been upgraded. The water's electricity is computed and updated from the sensor in Field 1. The pH level of the water source is being adjusted in Field 2. Water's pH is directly related to its voltage. It is given in Equation (2).

(2) E = E^0^ +(RT/zF) pH

Where E represents the cell potential under the current parameters, E^0^ denotes the cell's potential at normal pressure and temperature, R the common gas constant, and T the value of the temperature, z is the total amount of electrical units delivered to the response, while F denotes the rate constant Faraday. Different observation obtained in the experiment are provided in Table 6 and the respective graphical representation is shown in Fig. 12.

### 3.9 Turbidity sensor

Table 10 and Fig. 8 shows how the Server calculates and updates the turbidness measurements in the units called NTU and water's electricity (in volts). According to Eq. (3), the turbidity of water is inversely correlated to water's voltage.

(3) z = −1120.4y^2^+5742.3y–4352.9

In Eq. (3), ‘z’ represents the value of the turbidity and ‘y’ represents the voltage.

If the suggested system detects changes in environmental factors that exceed the threshold values, it will sound a warning via a buzzer. Environmental conditions significantly impact the reliability and performance of environmental monitoring systems. Extreme temperatures, humidity, pH levels, raindrop sensors, flood alarm sensors, turbidity sensors, and wind sensors can all affect their accuracy and stability. High temperatures can cause overheating, low temperatures can cause sensitivity, and humidity can cause corrosion. pH sensors require regular calibration and maintenance to extend their lifespan. Raindrop sensors should have high water saturation tolerance, sensitivity levels, fast response times, and durable construction. Rain gauge sensors should be robust, reliable, and calibrated to withstand high rainfall rates.

The proposed advanced real-time smart environmental parameters monitoring system offers several unique features and improvements compared to existing monitoring systems. The improvements are real-time monitoring of environmental parameters, wireless connectivity, data integration and analysis, advanced sensor technology, customizable alerting and reporting, scalability and flexibility, and a user-friendly interface. Therefore, overall, the proposed advanced real-time smart environmental parameters monitoring system offers enhanced capabilities. Also, the proposed method provides a comprehensive solution for monitoring and measuring multiple environmental parameters simultaneously, facilitating better environmental management and decision-making.

The effectiveness of a real-time smart environmental parameter monitoring system is determined by several aspects, including wireless connectivity range, sensor location, network architecture, geographical scope, scalability, and remote monitoring capabilities. Wireless communication can extend up to a few hundred feet indoors, and sensor placement assures optimal coverage. This Arduino Uno and ESP8266-based system can collect and send data from a variety of environmental sensors. The system's range and coverage are determined by the exact sensors utilized as well as the ESP8266 module's communication capabilities. The ESP8266 module, which provides Wi-Fi connectivity, has a typical range of approximately 100 m in an open area. Network infrastructure may limit coverage to locations with adequate network coverage. Geographical scope can be limited by physical location and deployment strategy. Scalability can be achieved by adding more sensors, microcontrollers, and communication infrastructure, but associated costs and logistics must be considered. The range and coverage of the system can also be influenced by power supply limitations and environmental constraints.

Real-time monitoring systems for environmental parameters can offer several cost-effective advantages compared to traditional monitoring methods. This system requires an upfront investment in equipment and infrastructure, but it can provide significant long-term cost savings compared to traditional monitoring methods. The ability to continuously collect accurate, immediate data, reduce labor costs, and make timely decisions based on real-time information can lead to improved efficiency and precision, risk mitigation, cost savings in maintenance and repair, scalability, and flexibility.

## 4 Conclusions

Everyone needs to be aware of the changes in the environment. Maintaining a positive atmosphere becomes important in enclosed and open workspaces like underground mines, office buildings, rooms, indoor, outdoor, or rooftop farms, seashores, mountains, and so on. Therefore, persistent observation of the environment employing real-time smart technology contributes to the collection of data required to recognize patterns, make estimations, and set variables, all of which are critical for early warning techniques. Accountability is one significant benefit of the growth of real-time monitoring of the environment. IoT has a significant impact on raising environmental standards. It has clever and cutting-edge methods for water treatment and air quality testing that significantly advance sustainable living. Hence, proposed an advanced real-time smart environmental parameters monitoring system using IoT and wireless sensors. That system captures real-time data through the use of several sensors which related sense the data of air, water, waste, energy and soil. The sensor network used here includes a temperature & humidity sensor, pressure sensor, gas sensor, air quality sensor, rain gauge, raindrop sensor, pH sensor, wind speed and direction sensors, soil moisture and light intensity. The sensed data is then sent to an Arduino Uno-based microcontroller, which communicates it via GSM, LCD, buzzer and Wi-Fi enabled HTTP based webpage. To facilitate data collecting and display, and to take advantage of technological improvements, a Wi-Fi module is used here to communicate the detected environmental parameters to a webpage where the recorded data can be viewed on an IoT-based mobile or personal computer. Perhaps the most beneficial IoT application is environmental monitoring. It promotes improved sustainability by using modern sensor devices to identify the presence of contaminants in the soil, waste, water, air, and energy. Using smart environmental monitoring technology can help you keep your premises safer and cleaner.

Implementing this IoT-based environmental parameter monitoring system can present a number of challenges and limitations, including connectivity issues, security and privacy concerns, scalability and interoperability, power constraints, data management and analytics, cost considerations, user experience, and adoption. Being aware of these challenges and restrictions allows you to plan and strategize accordingly, ensuring the successful installation of this IoT-based monitoring system.

Two important things to take into account for future work, first, the proposed architecture becomes more sophisticated when a greater number of sensors, communication, and alarm components are interconnected. The global positioning system (GPS) cannot be interfaced due to its complexity. Second, the proposed system's continually sensed data will be compared with data from forecasting agencies using various data analysis techniques to determine accuracy.

## Data availability statement

Data will be made available on request.

## Funding statement

Princess Nourah bint Abdulrahman University Researchers Supporting Project number (PNURSP2024R432), Princess Nourah bint Abdulrahman University, Riyadh, Saudi Arabia

## CRediT authorship contribution statement

**T. Lakshmi Narayana:** Writing – original draft, Project administration, Formal analysis, Data curation, Conceptualization. **C. Venkatesh:** Writing – review & editing, Formal analysis, Conceptualization. **Ajmeera Kiran:** Visualization, Validation, Project administration. **Chinna Babu J:** Supervision. **Adarsh Kumar:** Methodology, Formal analysis. **Surbhi Bhatia Khan:** Supervision, Funding acquisition. **Ahlam Almusharraf:** Writing – review & editing, supervision and validation. **Tabrez Quasim:** Writing – review & editing, Formal Analysis and validation.

## Declaration of competing interest

The authors declare that they have no known competing financial interests or personal relationships that could have appeared to influence the work reported in this paper.
